# A Diet Lacking Selenium, but Not Zinc, Copper or Manganese, Induces Anticancer Activity in Mice with Metastatic Cancers

**DOI:** 10.3390/nu16142249

**Published:** 2024-07-12

**Authors:** Patricia Díaz-Ortega, José Manuel Calderón-Montaño, Julio José Jiménez-Alonso, Emilio Guillén-Mancina, Víctor Jiménez-González, Estefanía Burgos-Morón, Miguel López-Lázaro

**Affiliations:** Department of Pharmacology, Faculty of Pharmacy, University of Seville, 41012 Sevilla, Spain

**Keywords:** cancer, metastasis, antitumor activity, ovarian cancer, breast cancer, colon cancer

## Abstract

Selenium, zinc, copper, and manganese are essential components of antioxidant enzymes involved in the elimination of reactive oxygen species (ROS). Given that cancer cells produce high levels of ROS and the accumulation of ROS can lead to cell death, cancer cells may be susceptible to strategies that reduce ROS elimination. In this work, we prepared several artificial diets that contained normal carbohydrate, protein, and lipid levels but lacked selenium, zinc, copper, or manganese. The anticancer activity of these diets was examined in a metastatic ovarian cancer model, established by injecting ID8 *Trp53*^−/−^ murine ovarian cancer cells into the peritoneal cavity of C57BL/6JRj mice. Treatments started 15 days later and consisted of replacing a normal diet with one of the artificial diets for several weeks. A significant improvement in mice survival was observed when the normal diet was replaced with the selenium-free diet. Diets lacking zinc, copper, or manganese showed no significant impact on mice survival. All diets were very well tolerated. The anticancer efficacy of a diet lacking selenium was confirmed in mice with metastatic colon cancer and in mice with metastatic triple-negative breast cancer. These results suggest that diets lacking selenium hold potential for the treatment of metastatic cancers.

## 1. Introduction

Despite advances in anticancer therapies, survival rates for patients with metastatic cancers remain low. For instance, the five-year relative survival rates for stage IV breast, prostate, ovarian, colon, lung, liver, and pancreatic cancers are 31%, 34%, 32%, 14%, 8%, 4%, and 3%, respectively [1]. Many patients who survive five years after diagnosis still succumb to the disease. A key reason for these low survival rates is the limited ability of current pharmacological treatments to selectively target cancer cells without harming normal cells. When treatment selectivity is suboptimal, oncologists often must administer the drugs at the maximum tolerated dose, rather than the effective dose, which is frequently insufficient for eradicating cancer cells. If oncologists were to administer the doses necessary to eradicate cancer cells, such doses would also have lethal effects on normal cells, ultimately leading to patient death. Identifying selective treatments could enhance cancer cell eradication and improve survival rates among patients with metastasis [2,3].

A potential strategy for achieving selective anticancer therapy involves upregulating the levels of reactive oxygen species (ROS) within cancer cells. It is well established that cancer cells produce high ROS levels relative to normal cells [4,5]. Evidence suggests that increasing ROS levels in cancer cells offers potential for targeted elimination, because the comparatively lower basal ROS levels in healthy cells serve as a safeguard against reaching toxic thresholds [4,5,6,7]. This anticancer strategy may be implemented by directly increasing ROS production or, alternatively, by decreasing ROS elimination capacity. Cancer cells use antioxidant enzymes, such as peroxidases, superoxide dismutase (SOD), and thioredoxin reductases, to eliminate ROS. These enzymes depend on essential minerals like selenium, zinc, copper, and manganese. One potential anticancer approach involves dietary deprivation of these micronutrients to suppress the enzymatic detoxification system, causing ROS accumulation in cells and selectively inducing cytotoxicity in cancer cells [5].

However, this anticancer approach might cause toxicity since these micronutrients have important physiological functions. For instance, selenium regulates several selenium-dependent enzymes known as selenoproteins. Unlike other enzymes regulated by micronutrients, selenoproteins incorporate selenium cotranslationally. The amino acid selenocysteine is integrated into elongating proteins during translation to form functional selenoproteins [8]. Examples of selenoproteins include glutathione peroxidases, thioredoxin reductases, iodothyronine deiodinases, and selenoprotein P. Glutathione peroxidases are key antioxidant enzymes; they play a crucial role in immune response, thyroid function, and reproductive processes in humans by coordinating antioxidant defense mechanisms [9,10,11,12]. Zinc, an essential micronutrient, is vital for many cellular processes such as growth, immune function, neurotransmission, vision, and reproduction. Over 3000 proteins in humans have zinc-binding sites, playing catalytic, structural, and regulatory roles. Catalytically, zinc supports many enzymes involved in numerous chemical reactions. For example, it catalyzes reactions through the enzyme Cu/Zn superoxide dismutase (Cu/Zn SOD), which detoxify superoxide anions. Structurally, zinc stabilizes proteins like zinc finger motifs, which are crucial for DNA binding and gene regulation. Metallothioneins, which bind zinc, help regulate metal ions, defend against oxidative stress, and detoxify heavy metals. Zinc also influences gene expression, cell signaling, hormone release, and nerve impulse transmission [13,14,15]. Copper plays a vital role in neutralizing free radicals and redox reactions. It is a component of cuproenzymes, including Cu/Zn SOD, which reduces superoxide anions to hydrogen peroxide (H_2_O_2_). In turn, other antioxidant enzymes such as glutathione peroxidases and iron-dependent catalase further reduce H_2_O_2_ to water, contributing to cellular antioxidant defense mechanisms [16,17,18,19]. Manganese plays a crucial role in the manganese superoxide dismutase (MnSOD) enzyme, a key mitochondrial antioxidant that converts superoxide radicals into hydrogen peroxide. MnSOD is also involved in several metabolic processes, including glucose and lipid metabolism, protein synthesis, skeletal growth, reproduction, and immune function [20]. The Recommended Daily Allowance (RDA) values for these micronutrients in different situations, along with the possible consequences of their deficiencies in humans, have been extensively reviewed by the Linus Pauling Institute’s Micronutrient Information Center [21]. The RDA of selenium is 55 μg/day for adults, the RDA for zinc is 8 mg/day for adult women and 11 mg/day for adult men, the RDA for copper is 900 μg/day for adults, and the RDA for manganese is 2.3 mg/day for adult men and 1.8 mg/day for adult women. The anticancer activity of selenium [22,23,24], zinc [25,26], copper [27,28,29], and manganese [24,30] has been reviewed previously. Several investigations have also shown that cancer patients frequently present with altered serum levels of these micronutrients, and these variations have been correlated with patient survival rates [31,32,33,34].

While sustained depletion of selenium, zinc, copper, or manganese may lead to toxicity, transient depletion could induce anticancer activity without toxic effects. Cancer cells, due to their higher ROS production, may rely more heavily on these micronutrients for antioxidant defense, making transient depletion of these elements a potential strategy to selectively induce cell death in cancer cells without harming normal cells. This study aims to evaluate the anticancer efficacy and tolerability of eliminating these micronutrients from the diet for several weeks. Our results demonstrate for the first time that a diet lacking selenium, but not zinc, copper, or manganese, increases the survival rates of mice with metastatic cancers, without causing toxicity in the animals.

## 2. Materials and Methods

### 2.1. Cell Culture and Drugs

CT26.WT (murine colorectal cancer cells, CRL-2638) and 4T1 (murine triple-negative breast cancer cells, CRL-2539), were obtained from the American Type Culture Collection (ATCC, Manassas, VA, USA). ID8 *Trp53*^−/−^ (murine ovarian cancer cells) were a gift from Dr. Iain A. McNeish (Institute of Cancer Sciences, University of Glasgow, Glasgow, UK) [35]. 4T1 and CT26.WT cells were cultured in RPMI 1640, which was supplemented with 100 U/mL penicillin, 100 μg/mL streptomycin, and 10% fetal bovine serum (FBS). ID8 *Trp53*^−/−^ were grown in Dulbecco’s modified Eagle’s medium (DMEM) high-glucose medium, which was supplemented with 0.11 g/L sodium pyruvate, 4% FBS, and 1% insulin–transferrin–selenium. All cells were cultured in a humidified 37 °C incubator with 5% CO_2_. Cisplatin was purchased from Thermo Scientific Acros Organics (Waltham, MA, USA), and capecitabine (500 mg/tablet, Normon, Madrid, Spain) was obtained from a local pharmacy (Seville, Spain). 

### 2.2. Animals

Female C57BL/6JRj mice (n = 70) and female BALB/cAnNRj mice (n = 50), 10 weeks or older, were obtained from Janvier Labs^®^ (Le Genest-Saint-Isle, France). To allow adequate acclimation, the mice were housed in our animal laboratory facilities for at least two weeks before the start of the experiments. The animals were maintained under standard conditions, including a 12 h light/12 h dark cycle, 70–75% humidity, and a temperature of 24 °C, with ad libitum access to food and water. The mice were provided with a standard diet (ssniff diet R/M-Z E/R/S; V1724-000, ssniff Spezialdiäten, Soest, Germany). At the beginning of the experiments, all mice were 12 weeks or older. The experiments were approved by the Animal Ethics Committee of the University of Seville (CEEA-US2018-6/2 and CEEA-US2019-20) and Junta de Andalucía (15/05/2018/090 and 13/11/2020/131) and were conducted following the recommendations of the European Union on animal experimentation (Directive of the European Council 2010/630/EU).

### 2.3. In Vivo Cancer Models

In the three cancer models used in this study, murine cells (9th–11th passage) were cultured in a 75 cm^2^ flask until reaching approximately 60–70% confluence. The medium was removed, and the cells were washed twice with sterile PBS. Then, the cells were incubated with trypsin/EDTA solution for 1–3 min at 37 °C, and the cells were suspended in 5 mL of sterile PBS. The cell suspension was pipetted up and down to break up any cell aggregates before adding 2.5% FBS-supplemented medium. A working cell suspension (ranging between 1 × 10^5^ and 5 × 10^6^ cells/mL, depending on the cancer model) was prepared. The cell suspension was centrifuged (250× *g*) at room temperature for 5 min, and the medium was removed. The cells were suspended in warm sterile filtered PBS and recounted to ensure the correct cell density. Finally, a 1 mL syringe was filled with 0.2 mL of the working cell suspension and injected into the peritoneal cavity or the tail vein of the mice.

One day before initiating the treatments, the mice were randomly assigned to different groups (randomization within blocks; the cage position on the racks was not randomized). The sample size was determined to ensure representative results, based on the experimental model and outcomes from previous experiments. Researchers were aware of group allocation during the experiments and data analysis. No injected mice were excluded unless specified. Untreated animals (control group) continued to receive their standard diet (ssniff diet). In the positive control group, mice were administered the maximum tolerated doses of an anticancer drug used in patients with the specific type of cancer under investigation (Table 1). For the groups of mice treated with the experimental diets, the treatment involved replacing their regular diet with one of the experimental diets. Depending on the model, treatments began four or fifteen days after injecting the cancer cells, allowing time for the cells to adapt to the new environment and proliferate before the start of the treatments. The objective of our research was not to prevent metastasis but to explore new treatments for patients with established metastatic cancers. The diet treatment lasted at least 6 weeks, unless otherwise specified. After the treatment period, the surviving animals returned to their normal diet. A summary of the three in vivo cancer models used in this study is provided in Table 1.

In the ovarian cancer model, 5 × 10^6^ ID8 *Trp53*^−/−^ cells were injected into the peritoneal cavity of female C57BL/6JRj mice [35]. Treatments started 15 days after the cancer cell inoculation. The positive control, cisplatin (5 mg/kg), was administered intraperitoneally once a week for 4 weeks.

The colon cancer model was established by injecting 10^5^ CT26.WT cells into the tail vein of female BALB/cAnNRj mice [36,37]. Treatments began 4 days after cancer cell inoculation. Capecitabine (450 mg/kg/day) was employed as a positive control and was administered in the diet following a schedule of 7 days on and 7 days off (7/7) to maximize the anticancer activity of this drug [38]. The animals received 2 or 3 cycles depending on their state of health at the moment of initiating the third capecitabine cycle. To prepare the capecitabine treatment, we powdered the standard diet (ssniff diet) and added capecitabine at a dose of 2500 mg/kg of diet; then, we added water to make a soft dough, which was pelleted and left to air-dry until use. A 25 g mouse typically consumed an average of 4.5 g of diet per day, resulting in a capecitabine dose of approximately 450 mg/kg/day.

In the triple-negative breast cancer model, female BALB/cAnNRj mice were inoculated with 10^5^ 4T1 cancer cells in the tail vein [39,40]. Treatments were initiated 4 days after the injection of the cells. The positive control, cisplatin (5 mg/kg), was administered intraperitoneally once a week for 4 weeks.

In the three models, all animals underwent daily monitoring, with body weights measured at least three times a week. Mice were humanely euthanized via cervical dislocation when signs of disease progression became evident. These signs included substantial fluctuations in body weight, diminished mobility and curiosity, respiratory distress, and/or the presence of visible or palpable tumors exceeding 15–20 mm. These indicators suggested that survival for an additional 2–3 days was unlikely. A postmortem examination was conducted to visualize the presence of tumors, assess the extent of the disease, and weigh and observe selected organs. Unless specified otherwise, autopsies consistently confirmed the presence of tumors and similar tumor loads in all euthanized mice. The detailed clinical signs and symptoms used to determine when to sacrifice mice, along with photographs illustrating tumor locations and loads observed in these cancer models, have been previously shown [41]. 

### 2.4. Diet Preparation and Composition

The six artificial diets used in this study were prepared in our laboratory by blending all solid components until they formed a homogeneous dry powder. Next, oil was incorporated into the mixture, and enough water was gradually added to achieve a soft dough consistency. This dough was air-dried for approximately 2 h; pellets were manually formed (approximately 5 g per pellet), further air-dried for an additional 24–48 h, and then stored at room temperature until required. Table 2 shows the composition of the diets employed in our study.

Casein (bovine casein 27607) was obtained from Acros Organics (Waltham, MA, USA). Sucrose and corn oil were purchased from a local market (MAS Supermarket, Seville, Spain). Cellulose and corn starch were bought from Farmusal (local pharmacy, Granada, Spain). Choline bitartrate (450225000) and *tert*-butylhydroquinone (150822500) were purchased from Thermo Scientific Acros Organics (Waltham, MA, USA). The dry diets contained 1% Vitamin Mix (AIN Vitamin Mixture 76, MP Biomedical, Seven Hills, Australia). A total of 100 g of the dry diets contained (mg) thiamine hydrochloride (0.6), riboflavin (0.6), pyridoxine hydrochloride (0.7), nicotinic acid (3), d-calcium pantothenate (1.6), folic acid (0.2), d-biotin (0.02), cyanocobalamin (0.001), retinyl palmitate premix (250,000 IU/g) (1.6), DL-α-tocopherol acetate (250 IU/g) (20), cholecalciferol (400,000 IU/g) (0.25), and menaquinone (0.005). A total of 1g of Vitamin Mix contained 0.9729 g of sucrose. Our Mineral Mix contained potassium phosphate monobasic (Budenheim, Budenheim, Germany), sodium fluoride (Fagron, Terrassa, Spain), iron (III) citrate (Dr. Paul Lohmann, Emmerthal, Germany), calcium carbonate (Dr. Paul Lohmann, Emmerthal, Germany), boric acid (Nutrifoods, Barcelona, Spain), potassium sulphate (Dr. Paul Lohmann, Emmerthal, Germany), potassium iodate (Nutrifoods, Barcelona, Spain), magnesium oxide (Nutrifoods, Barcelona, Spain), sodium chloride (Nutrifoods, Barcelona, Spain), silicon dioxide (Azelis, Barcelona, Spain), potassium citrate monohydrate (Nutrifoods, Barcelona, Spain), ammonium molybdate (Quality Chemicals S.L., Barcelona, Spain), chromium (III) chloride hexahydrate (Cambridge Commodities, Ely, UK), Nickel (II) carbonate hydroxide tetrahydrate (Sigma-Aldrich, Darmstadt, Germany), ammonium metavanadate (Sigma-Aldrich, Darmstadt, Germany), and lithium chloride (Sigma-Aldrich, Darmstadt, Germany). Our experimental diets (Table 2) also contained sodium selenate (Quality Chemicals S.L., Barcelona, Spain), manganese carbonate (Dr. Paul Lohmann, Emmerthal, Germany), basic copper carbonate (Dr. Paul Lohmann, Emmerthal, Germany), and/or basic zinc carbonate (Fagron, Terrassa, Spain). The ssniff diet was employed as a standard control diet (SM R/M-S E, 10 mm; V1724-000). This diet comprises 21% protein, 7% fat, 4% fiber, 6.2% ash, 33.3% starch, and 4.6% sugar. Specific components of the ssniff diet and their percentages, shown in brackets, are as follows: lysine (1.66), methionine (0.91), cystine (0.38), threonine (0.76), tryptophan (0.26), arginine (1.27), histidine (0.51), valine (0.98), isoleucine (0.89), leucine (1.57), phenylalanine (0.99), glycine (0.88), tyrosine (0.70), glutamic acid (4.38), aspartic acid (1.99), proline (1.41), serine (1.09), alanine (0.96), vitamin A (2500 IU), vitamin D3 (150 IU), vitamin E (0.0135), vitamin K (0.008), thiamine (0.0085), riboflavin (0.0032), pyridoxine (0.0031), cobalamin (0.000015), nicotinic acid (0.014), pantothenic acid (0.0059), folic acid (0.001), biotin (0.000069), choline chloride (0.337), ferrous sulphate monohydrate (0.01), zinc sulphate monohydrate (0.005), manganous-(II)-sulphate monohydrate (0.003), copper-(II)-sulfate pentahydrate (0.0005), sodium selenate (0.00001), and calcium iodate anhydrous (0.0002).

### 2.5. Statistical Analysis

Results are presented as mean ± standard error of the mean (SEM). Statistical analysis was conducted using GraphPad Prism (Boston, MA, USA) version 7.0 software. The Kaplan–Meier survival curve was subjected to statistical analysis using the Gehan–Breslow–Wilcoxon (GBW) test. A *p* value > 0.05 is considered not statistically significant and is not denoted by any symbol. A *p* value < 0.05 is considered statistically significant and is indicated with an asterisk (*), <0.01 with two asterisks (**), and <0.001 with three asterisks (***).

## 3. Results

### 3.1. A Diet Lacking Selenium, but Not Zinc, Copper, or Manganese, Induces Anticancer Activity in Mice with Metastatic Ovarian Cancer

To evaluate whether the dietary deprivation of selenium, zinc, copper, and manganese induces anticancer activity in mice with metastatic cancers, we employed an ovarian cancer model. The model was established by inoculating murine ID8 *Trp53*^−/−^ ovarian cancer cells into the peritoneal cavity of female C57BL/6JRj mice. Treatments started 15 days after the inoculation of the cancer cells. The positive control, cisplatin (5 mg/kg), was administered intraperitoneally once a week for four weeks. We used a standard rodent diet (“Normal diet”) for the control group. We prepared six diets from scratch: a control diet that contained normal levels of selenium, zinc, copper, and manganese (control, C), and five diets that lacked selenium (C-Se), zinc (C-Zn), copper (C-Cu), manganese (C-Mn), or the four micronutrients (C-SZCM) (see Table 2). Mice treated with cisplatin were fed the standard rodent diet. 

**Figure 1:**
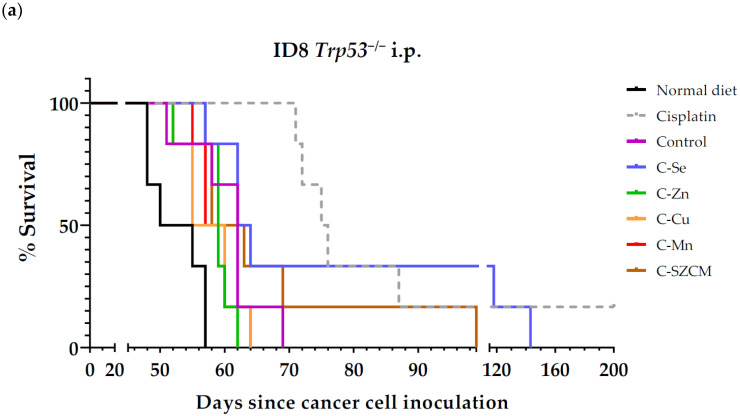

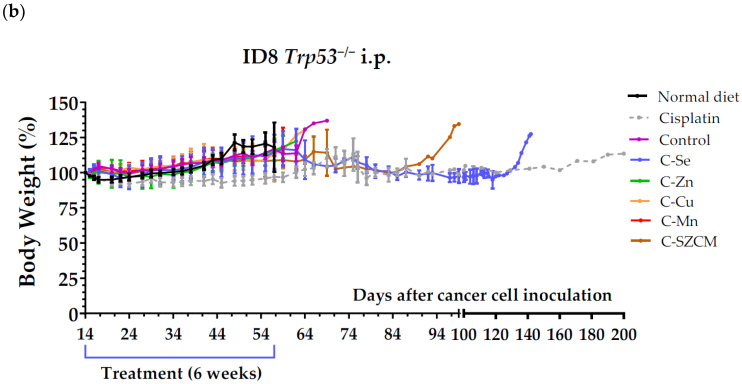
Anticancer activity of cisplatin and diets lacking selenium, zinc, copper, or manganese in mice with metastatic ovarian cancer (ID8 *Trp53*^−/−^ murine ovarian cancer cells inoculated in peritoneal cavity of female C57BL/6JRj mice). (**a**) Survival of mice treated with cisplatin (intraperitoneal administration of 5 mg/kg cisplatin once a week for 4 weeks), mice fed normal standard diet, control diet prepared from scratch, and diets lacking selenium, zinc, copper, and/or manganese. (**b**) Body weights of mice relative to body weights at beginning of treatments (day 15). Normal diet: standard rodent diet; control: control diet prepared from scratch; C-Se: diet without selenium; C-Zn; diet without zinc; C-Cu: diet without copper; C-Mn: diet without manganese; C-SZCM: diet without selenium, zinc, copper, and manganese.

As presented in Table 3 and Figure 1a, the selenium-free diet enhanced the survival of mice with metastatic ovarian cancer. Mice fed the standard rodent diet lived 52.5 ± 1.6 days, mice fed the control diet prepared from scratch lived 60.7 ± 2.2 days, while mice fed the selenium-free diet lived 84.3 ± 13.7 days. Eliminating selenium from the diet improved mice survival by over 3 weeks (23.6 days). Mice fed our control diet prepared from scratch lived approximately 8 days longer than those receiving the standard rodent diet. Since our control diet contained casein, a protein with low levels of the sulfur amino acids (AAs) methionine and cysteine, the increased survival might be attributed to the deficiency in these AAs [42]. Mice treated with diets lacking zinc, copper, or manganese had slightly shorter survival times (non-statistically significant) compared to those fed the control diet. The mean ± SEM survival time was 58.5 ± 1.3 days for mice fed diet C-Zn, 57.7 ± 1.6 days for mice fed diet C-Cu, and 58.7 ± 1.2 days for mice fed diet C-Mn. Mice fed diet C-SZCM exhibited a moderate increase (non-statistically significant) in survival (67.3 ± 6.0 days). Cisplatin-treated mice demonstrated the highest survival rate (>96.8 ± 19.0 days). One mouse treated with cisplatin was apparently cured; it was sacrificed on day 200 without showing any sign of cancer or tumors on autopsy. Cisplatin administration into the peritoneal cavity may partially account for its significant anticancer activity because the ovarian cancer cells were also inoculated in the same place. All artificial diets were well tolerated, with no significant changes in body weights (Figure 1b). The weight gain observed in some groups prior to sacrifice can be attributed to ascitic tumor growth and the characteristic intraperitoneal fluid accumulation in this model.

**Figure 2:**
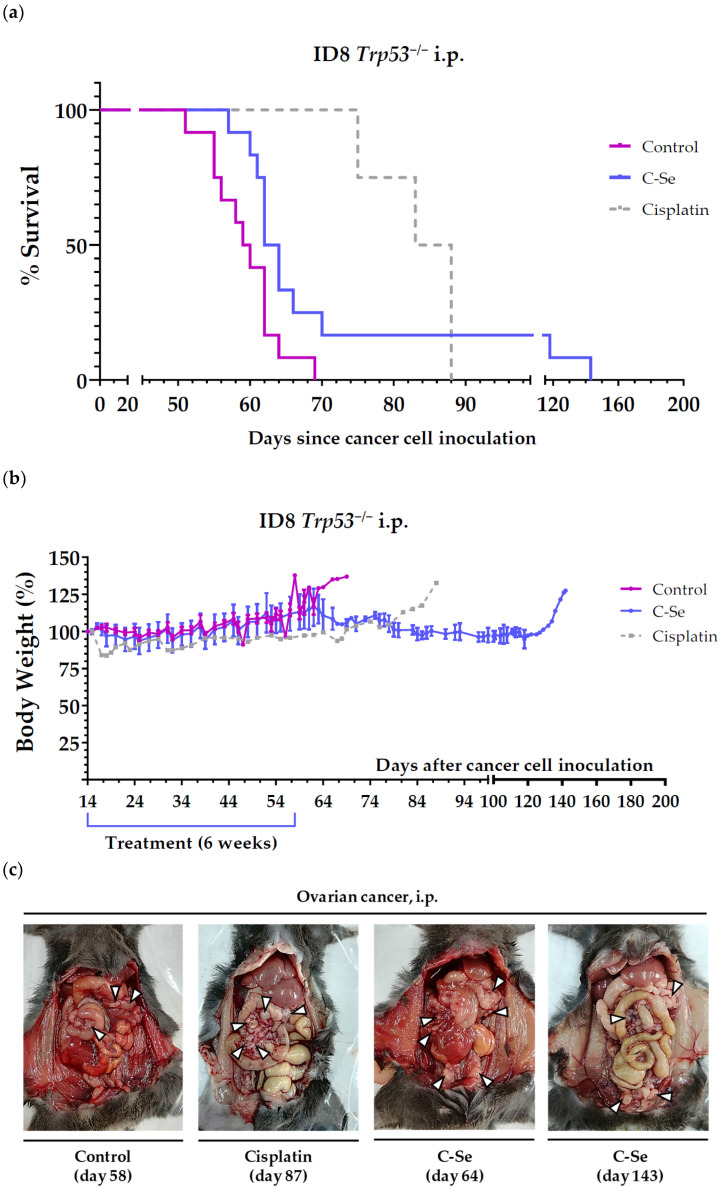
The anticancer activity of cisplatin and a diet lacking selenium in mice with metastatic ovarian cancer (stablished by inoculating ID8 *Trp53*^−/−^ murine ovarian cancer cells in the peritoneal cavity of female C57BL/6JRj mice). (**a**) The survival of mice fed a control diet prepared from scratch (control), mice fed a diet lacking selenium (C-Se), and mice fed the control diet and treated with cisplatin (intraperitoneal administration of 5 mg/kg once a week for 4 weeks). (**b**) The body weights of the mice relative to their body weights at the beginning of treatments (day 15). (**c**) Representative photographs at the time of sacrifice of the peritoneal cavity of mice with ovarian cancer. White arrowheads point to specific tumors. The day of sacrifice is shown in brackets. Data for the control and C-Se groups are from two independent experiments (see text for details).

We next employed this ovarian cancer model to assess the anticancer efficacy of a diet lacking selenium in combination with cisplatin. Mice survival (mean ± SEM) was 58.2 ± 1.4 days in mice fed the C diet (control diet prepared from scratch), 63.8 ± 1.5 days in mice fed the C-Se diet (selenium-free diet), 83.5 ± 3.1 days in mice receiving the C diet and cisplatin, and 84.3 ± 5.0 days in mice receiving the C-Se diet and cisplatin. The combination of cisplatin and the selenium-free diet did not significantly improve mice survival. In this second experiment, none of the animals receiving the selenium-free diet had a major improvement in survival. Table 4 and Figure 2a show the survival time of all mice with metastatic ovarian cancer treated with the selenium-free diet (obtained from the two independent experiments carried out in this model). Eliminating selenium from the diet significantly increased mice survival by 14.7 days. The diet was very well tolerated, and no significant weight losses were observed (Figure 2b).

### 3.2. A Diet Lacking Selenium Induces Anticancer Activity in Mice with Metastasic Colon Cancer

**Figure 3:**
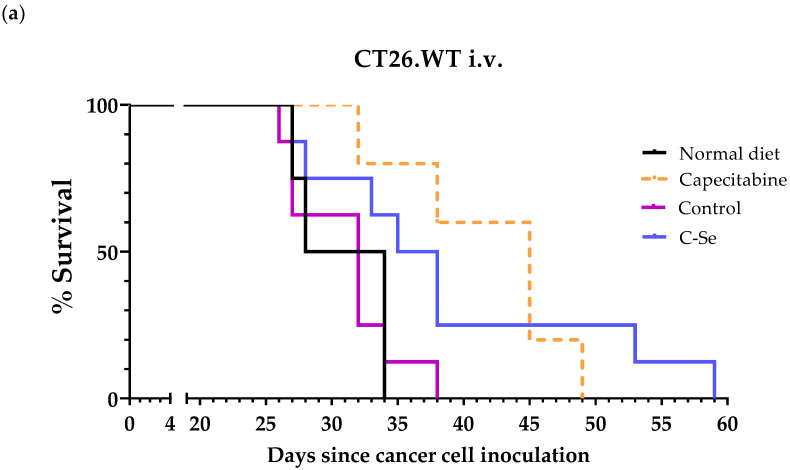

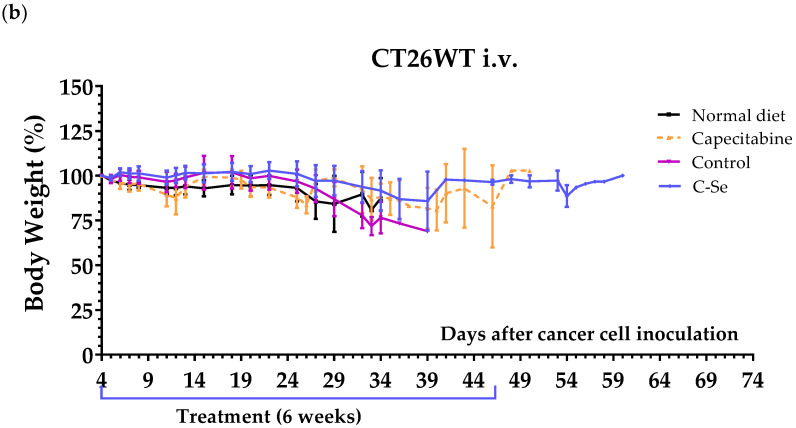
The anticancer activity of capecitabine and a diet lacking selenium in mice with metastatic colon cancer (CT26.WT cells inoculated in the tail vein of female BALB/cAnNRj mice). (**a**) The survival of mice treated with capecitabine (450 mg/kg/day; administered in the diet following a 7 days on/7 days off schedule for 2 or 3 cycles depending on their state of health), mice fed a normal standard diet, a control diet prepared from scratch, and a diet lacking selenium. (**b**) The body weights of the mice relative to their body weights at the beginning of treatments (day 4). Normal diet: standard rodent diet; control: control diet prepared from scratch; C-Se: diet without selenium.

To confirm the efficacy of the selenium-free diet in mice with other metastatic cancers, we used a metastatic colon cancer model. Treatments were initiated four days after the inoculation of CT26.WT cells into the tail vein of immunocompetent female BALB/cAnNRj mice. Capecitabine (450 mg/kg/day), a first-line drug for metastatic colon cancer, served as a positive control. Mice on the standard rodent diet lived 30.0 ± 1.5 days, and those on the control diet lived 31.0 ± 1.4 days. The selenium-free diet extended survival to 38.9 ± 3.8 days, and capecitabine (mixed in the standard rodent diet) treatment resulted in 41.8 ± 2.7 days of survival (Table 5, Figure 3a). The selenium-free diet was very well tolerated and did not induce significant weight loss in the animals; however, moderate weight loss was observed in the capecitabine group (Figure 3b). Mice administered capecitabine also exhibited reductions in spontaneous motor activity, which reversed after the completion of each treatment.

### 3.3. A Diet Lacking Selenium Induces Anticancer Activity in Mice with Metastasic Triple-Negative Breast Cancer

**Figure 4:**
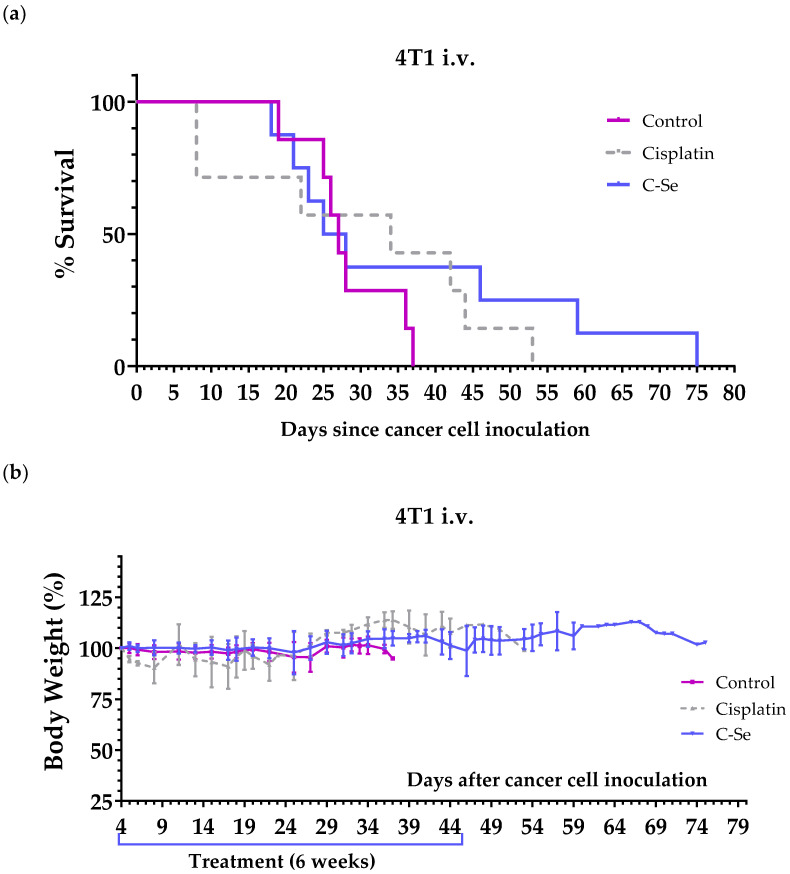
The anticancer activity of cisplatin and a diet lacking selenium in mice with metastatic triple-negative breast cancer (4T1 murine breast cancer cells inoculated in the tail vein of female BALB/cAnNRj mice). (**a**) The survival of mice treated with cisplatin (intraperitoneal administration of 5 mg/kg once a week for 4 weeks), mice fed a control diet prepared from scratch, and a diet lacking selenium. (**b**) The body weights of the mice relative to their body weights at the beginning of treatments (day 4). Control: control diet prepared from scratch; C-Se: diet without selenium.

We used a murine metastatic triple-negative breast cancer model to further validate the anticancer activity of the selenium-free diet. This model was established by inoculating murine 4T1 cells in the tail vein of female BALB/cAnNRj mice. Treatments started 4 days after the injection of the cells. The positive control cisplatin (5 mg/kg) was administered intraperitoneally once a week for 4 weeks. Mice on the control diet lived 28.3 ± 2.2 days, while those on the selenium-free diet lived 36.9 ± 6.9 days. Cisplatin treatment resulted in 39.0 ± 4.7 days of survival (Table 6, Figure 4a). The diet lacking selenium was very well tolerated, and no weight loss was observed (Figure 4b). In contrast, moderate weight loss was observed in most mice treated with cisplatin, and two mice treated with this drug died on day 8 because of cisplatin toxicity. These two mice started to lose weight and developed other signs of toxicity (e.g., abnormal gait/posture, pale ears, lack of curiosity, and reluctance to move) after the first cisplatin injection (these two mice are not included in Table 6 but are shown in Figure 4a).

## 4. Discussion

It is widely accepted that patients with non-resectable metastatic cancers must be treated with drugs. The objective of this work is to evaluate a new therapeutic strategy for the treatment of metastatic cancers that does not rely on the use of drugs. Instead of administering cytotoxic molecules to kill cancer cells, our strategy consists of temporarily eliminating dietary components that cancer cells may need to survive. Selenium, zinc, copper, and manganese are essential components of antioxidant enzymes involved in the elimination of reactive oxygen species (ROS). Since cancer cells produce high levels of ROS and ROS accumulation induces cell death, cancer cells may be vulnerable to diets lacking these micronutrients. In this work, we prepared several artificial diets containing normal carbohydrate, protein, and lipid levels but lacking selenium, zinc, copper, or manganese and evaluated their anticancer activity in mice with metastatic cancers.

To test the hypothesis that dietary depletion of these micronutrients can induce anticancer activity in mice with metastatic cancer, we initially used an ovarian cancer model. Our initial experiment showed that the selenium-free diet enhanced the mean survival of mice with disseminated ovarian cancer, whereas diets lacking zinc, copper, or manganese did not exhibit anticancer activity (Table 3, Figure 1). The diet deficient in all four micronutrients (C-SZCM) showed less anticancer activity compared to the diet lacking selenium only (Table 3). Although previous studies indicate that zinc, copper, and manganese deficiencies can increase ROS levels and induce oxidative stress [43,44], our diets lacking zinc, copper, or manganese failed to exhibit anticancer activity. The accumulation of H_2_O_2_ might explain why selenium restriction induces anticancer activity, in contrast to deficiencies in zinc, copper, and manganese. Cancer cells are known to produce high levels of H_2_O_2_ [4] and the cellular accumulation of H_2_O_2_ can selectively kill cancer cells [5,45]. Copper, zinc, and manganese are essential components of SOD enzymes, which convert superoxide anions into H_2_O_2_. Later, selenium-dependent enzymes (glutathione peroxidases and thioredoxin reductases) and selenium-independent enzymes (catalase and peroxiredoxins) can convert H_2_O_2_ into water [5,7]. Consequently, diets lacking copper, zinc, or manganese would reduce H_2_O_2_ production, while diets lacking selenium would result in H_2_O_2_ accumulation. The diet lacking all four minerals would reduce the activity of SOD enzymes, thereby decreasing the conversion of superoxide anions into H_2_O_2_. Simultaneously, selenium deficiency would reduce the function of selenium-dependent enzymes responsible for eliminating H_2_O_2_, which would lead to an accumulation of H_2_O_2_ that is produced by other sources that do not require SOD activity. The net effect would depend on the balance between reduced H_2_O_2_ production (due to copper, zinc, and manganese deficiencies) and impaired H_2_O_2_ detoxification (due to selenium deficiency). The mild survival improvements observed in mice fed the diet lacking the four minerals suggest that the selenium deficiency-driven H_2_O_2_ accumulation might still be high enough to kill a small percentage of cancer cells and increase the mean survival of the mice. Accordingly, previous data from a non-metastatic xenograft model of glioblastoma showed that selenium deficiency significantly reduced circulating selenium levels and increased the anticancer activity of ascorbate by inducing H_2_O_2_ accumulation [46]. 

We conducted an additional experiment to confirm that the selenium-free diet improved the survival of mice with ovarian cancer. Although we observed improved survival rates compared to untreated mice, mice treated with the standard anticancer agent cisplatin exhibited longer survival benefits (Table 3 and Table 4, Figure 1 and Figure 2). The high efficacy of cisplatin might be partially attributed to the direct injection of this drug into the region where ovarian cancer cells were inoculated in mice. The combination of cisplatin with the selenium-free diet did not significantly enhance the survival observed with cisplatin alone. These data suggest that the concomitant use of cisplatin therapy and a diet lacking selenium would not result in significant improvements in the survival of patients with ovarian cancer, at least under our experimental conditions.

To further validate the anticancer activity of the selenium-free diet, we employed a metastatic model of colon cancer, established by inoculating murine colon cancer cells into the tail vein of immunocompetent BALB/cAnNRj mice. Mice fed the selenium-free diet lived approximately 9 days longer than mice fed a standard rodent diet, which was 3 days shorter than the survival achieved in the group of mice treated with capecitabine (Table 5, Figure 3). Capecitabine is a first-line anticancer drug widely used for treating people with metastatic colon cancer. Notably, unlike mice treated with capecitabine, those fed the selenium-free diet did not exhibit any signs of toxicity.

Lastly, we assessed the anticancer activity of the selenium-free diet in a metastatic model of triple-negative breast cancer, established by injecting 4T1 murine cancer cells into the tail vein of immunocompetent BALB/cAnNRj mice. Our findings revealed enhanced mean survival in mice treated with the selenium-deficient diet compared to the control group, with efficacy similar to that observed in the cisplatin-treated group. The selenium-free diet was well tolerated, with no observed weight loss (Figure 4b). In contrast, cisplatin induced moderate weight loss in most mice, and two mice treated with this drug died on day 8 due to cisplatin toxicity (Table 6, Figure 4).

While our study is the first to investigate the effects of dietary selenium removal in mice with metastatic cancers, earlier in vivo research has shown that selenium deficiency can prevent cancer formation and delay the growth of localized tumors in mice. Felix et al. [47] found that all BALB/c mice maintained on a torula-based low-selenium diet (5–8 µg of selenium/kg) were completely resistant to pristane induction of peritoneal plasmacytomas. In contrast, 42.3% of control mice on a selenium-adequate torula diet (300 µg of selenium/kg) and 37.5% of control mice fed standard Purina chow (440 µg of selenium/kg) developed peritoneal plasmacytomas [47]. A diet lacking selenium also reduced tumor incidence in TGFα/c-Myc transgenic mice; these mice have disrupted redox homeostasis and develop liver cancer by 6 months of age [48]. Selenium dietary restriction also induced anticancer activity and improved ascorbate efficacy in a mouse xenograft model of glioblastoma [46]. Selenium dietary restriction has also been shown to alleviate leukocytosis, decrease leukemic burden, and significantly prolong leukemia-free survival in xenograft models of acute myeloid leukemia (AML). Notably, these effects occurred with minimal impact on normal hematopoiesis [49].

Consistent with our findings, earlier studies have shown that removing copper and zinc from the diet does not exhibit anticancer activity. In a study involving male Sprague-Dawley rats fed copper-deficient and -non-deficient diets (7 days before cancer cell implantation), it was concluded that copper deficiency had no impact on tumor growth, vascular density, or ultrastructural tumor characteristics [50]. Additionally, dietary zinc deficiency was found to increase the incidence and reduce the lag time for the induction of esophageal tumors in rats by methylbenzylnitrosamine [51]. Although diets lacking copper have not shown anticancer activity, copper chelators have shown antitumor potential due to their ability to disrupt copper homeostasis in cancer cells. By binding to and removing excess copper, these chelators can inhibit tumor growth and metastasis [52,53].

It is worth noting that our selenium-free diet improved mean survival in mice with three distinct types of metastatic cancers (ovarian, colon, and breast cancer) without causing toxicity in the animals. Triple-negative breast cancer (TNBC) is known to be a particularly aggressive form of breast cancer [54,55], and our metastatic TNBC mouse model is particularly challenging. Indeed, we previously observed that two drugs commonly used to treat women with metastatic TNBC, capecitabine and doxorubicin, were virtually ineffective in our model [41]. While cisplatin enhanced mice survival in this model, two mice died on day 8 due to cisplatin toxicity (Table 6, Figure 4). Given that the only difference between our control diet and the selenium-free diet is the presence or absence of a small amount of selenium (Table 2), we can conclude that the dietary elimination of selenium for several weeks can improve the survival rates of mice with different types of metastatic cancers. Although our artificial diets were formulated without Se, Zn, Cu, and/or Mn, the final levels of these micronutrients in the diets given to the animals have not been analyzed. Since we used some dietary components that are not chemically defined (e.g., casein), we cannot assure that the final amount of Se in our C-Se diet is exactly 0%. 

The lack of toxicity of our selenium-free diet may seem surprising, given that selenium is an essential micronutrient with crucial physiological roles. However, evidence suggests that adult individuals can tolerate selenium deficiency for extended periods. Yim et al. [56] reported that, while a selenium-free diet led to a significant decline in selenoprotein expression, mice subjected to this dietary regimen throughout their life had normal lifespans. To understand the underlying molecular mechanisms, the authors conducted systemic analyses at the levels of metabolome, transcriptome, and microRNA profiling. Their findings indicated that selenium deficiency reduced amino acid levels, elevated mononucleotides, altered metabolism, and activated signaling pathways associated with longevity-related nutrient sensing. These activated pathways appear to protect mice against the absence of dietary selenium. Furthermore, the study confirmed the known priority for selenium supply in different organs; the liver showed a rapid depletion of selenoproteins in response to selenium deficiency, whereas brain selenoprotein expression remained largely unchanged [56]. In humans, 99 days on a low-selenium diet did not change the overall health of a small group of men [57].

Several mechanisms may contribute to explaining the selective anticancer activity of selenium deficiency in vivo. As previously mentioned, selenium plays a critical role in the detoxification of H_2_O_2_. Since cancer cells produce substantial amounts of H_2_O_2_ [4,5], they may require higher selenium levels than normal cells to prevent the accumulation of cytotoxic H_2_O_2_ concentrations. Consistently, a radioactive form of dietary selenium compound (selenite) was found to accumulate preferentially in tumors, a phenomenon explored as a tumor-labeling strategy in clinical settings during the 1960s [58,59]. More recently, Charalabopoulos et al. [34] conducted a study involving 80 women with breast cancer who underwent radical mastectomy. The results revealed that serum selenium levels were 42.5 ± 7.5 µg/L in breast cancer patients, compared to 67.6 ± 5.36 µg/L in an age-matched control group of healthy individuals. However, selenium concentrations in neoplastic tissues were 2660 ± 210 mg/g tissue, whereas those in adjacent non-neoplastic tissues were 680 ± 110 mg/g tissue (*p* < 0.001). These data demonstrate that, despite lower serum selenium levels in women with breast cancer compared to healthy individuals, selenium concentrations in breast cancer tissues were significantly higher than in adjacent non-cancerous tissues. Carlisle et al. [60] demonstrated that selenophosphate synthetase 2 (SEPHS2), an enzyme in the selenocysteine biosynthesis pathway (essential for the production of all selenoproteins), was vital for the survival of many cancer cell types but not for normal cells. Non-transformed MCF10A cells lacking SEPHS2 were able to maintain their growth rate for 28 days despite losing SEPHS2 and selenoprotein expression capabilities [60]. The authors suggested that, under basal conditions, normal cells neither require SEPHS2 nor selenoprotein production for their survival and proliferation. They also observed that cancer cells can activate the cystine/glutamate antiporter SLC7A11 to promote selenium uptake [60]. Eagle et al. [49] reported that SEPHS2 regulates selenoprotein production in AML, and its suppression through diet renders these leukemia cells susceptible to oxidative stress without harming hematopoiesis [49]. Reduced amino acid levels caused by selenium-free diets may also contribute to explaining the selective anticancer activity of selenium deficiency in vivo. Yim et al. [56] found that selenium deficiency significantly reduced the levels of seven amino acids: asparagine, leucine, lysine, methionine, phenylalanine, serine, and threonine. Amino acid restriction can induce selective killing of cancer cells in vitro and improve the survival rates of mice with several metastatic cancer types, as reviewed in [61].

Although our selenium-free diet enhanced the survival of mice with several metastatic cancers without causing toxicity, it is important to note that all mice eventually died. In our previous studies [41,42,62,63], we found that several artificial diets with modified amino acid and lipid levels led to significant improvements in the survival rates of mice with several metastatic cancers. Some diets even outperformed standard anticancer therapies and cured a small percentage of mice [41,42,62,63]. Currently, we are evaluating the anticancer activity of a variety of selenium-free diets with altered amino acid and lipid levels in animal models of metastasis. We are also testing one of these selenium-free diets as monotherapy in patients with advanced metastatic cancers. Their blood samples are being analyzed at various time points to measure various parameters, including selenium levels. Although these artificial diets could be clinically useful as monotherapy, we are also testing their efficacy in mice in combination with standard drugs used in cancer patients.

## 5. Conclusions

Our findings demonstrate for the first time that a selenium-free diet, but not diets lacking zinc, copper, or manganese, enhances the mean survival rates of mice with metastatic cancers, without causing toxicity in the animals. These results underscore the potential of using selenium-free diets in the treatment of metastatic cancers. Further research is warranted to explore potential mechanisms and optimal dietary strategies to maximize the anticancer efficacy of selenium-free diets, both as a monotherapy and in combination with existing cancer therapies. 

## Figures and Tables

**Table 1:** In vivo metastatic cancer models.

Cancer Model	Metastatic Localization	Cell Line Inoculation	Mice (Sex, Train)	Positive Control Drug	Treatment Start Day
Ovarian cancer	Peritoneal dissemination	5,000,000 ID8 *Trp53*^−/−^ cells into the peritoneal cavity	Female C57BL/6JRj	Cisplatin 5 mg/kg i.p. once a week for 4 weeks	15
Colon cancer	Pulmonary metastases	100,000 CT26.WT cells into the tail vein	Female BALB/cAnNRj	Capecitabine 450 mg/kg/day in the diet 7/7 on/off schedule	4
Triple-negative breast cancer	Pulmonary metastases	100,000 4T1 cells into the tail vein	Female BALB/cAnNRj	Cisplatin 5 mg/kg i.p. once a week for 4 weeks	4

**Table 2:** The composition of the six experimental diets (g/100 g dry diet).

DIET	C	C-Se	C-Zn	C-Cu	C-Mn	C-SZCM
Casein	20	20	20	20	20	20
Corn oil	7	7	7	7	7	7
Corn starch	48.498	48.498	48.498	48.498	48.498	48.498
Sucrose	15	15	15	15	15	15
Cellulose	5	5	5	5	5	5
Choline bitartrate	0.25	0.25	0.25	0.25	0.25	0.25
Vitamin mix	1	1	1	1	1	1
Tert-butylhydroquinone	0.0008	0.0008	0.0008	0.0008	0.0008	0.0008
Calcium carbonate	1.00	1.00	1.00	1.00	1.00	1.00
Potassium phosphate monobasic	0.88	0.88	0.88	0.88	0.88	0.88
Potassium citrate monohydrate	0.098	0.098	0.098	0.098	0.098	0.098
Sodium chloride	0.26	0.26	0.26	0.26	0.26	0.26
Potassium sulphate	0.16	0.16	0.16	0.16	0.16	0.16
Iron (III) citrate	0.028	0.028	0.028	0.028	0.028	0.028
Potassium iodate	0.000051	0.000051	0.000051	0.000051	0.000051	0.000051
Ammonium molybdate	0.000025	0.000025	0.000025	0.000025	0.000025	0.000025
Silicon dioxide	0.0011	0.0011	0.0011	0.0011	0.0011	0.0011
Chromium (III) chloride hexahydrate	0.00090	0.00090	0.00090	0.00090	0.00090	0.00090
Boric acid	0.00029	0.00029	0.00029	0.00029	0.00029	0.00029
Magnesium oxide	0.084	0.084	0.084	0.084	0.084	0.084
Sodium fluoride	0.00022	0.00022	0.00022	0.00022	0.00022	0.00022
Nickel (II) carbonate hydroxide tetrahydrate	0.00011	0.00011	0.00011	0.00011	0.00011	0.00011
Ammonium metavanadate	0.000023	0.000023	0.000023	0.000023	0.000023	0.000023
Lithium chloride	0.000061	0.000061	0.000061	0.000061	0.000061	0.000061
Basic copper carbonate	0.0011	0.0011	0.0011	0	0.0011	0
Basic zinc carbonate	0.0055	0.0055	0	0.0055	0.0055	0
Manganese carbonate	0.0024	0.0024	0.0024	0.0024	0	0
Sodium selenate	0.000036	0	0.000036	0.000036	0.000036	0

C: control diet, C-Se: diet lacking selenium, C-Zn; diet lacking zinc, C-Cu: diet lacking copper, C-Mn: diet lacking manganese, C-SZCM: diet lacking selenium, zinc, copper, and manganese.

**Table 3:** Survival of mice with ovarian cancer treated with cisplatin or with diets lacking selenium, zinc, copper, manganese, or the four micronutrients.

Treatment	n	Survival Time (Mean ± SEM; Days)	Survival Improvement vs. Control (Days)	*p* Value vs. Control
Normal diet	6	52.5 ± 1.6	-	-
Cisplatin (a)	6	>96.8 ± 19.0	>+44.3	0.0015 (**)
Control (C)	6	60.7 ± 2.2	-	-
C-Se	6	84.3 ± 13.7	+23.6	0.2466
C-Zn	6	58.5 ± 1.3	−2.2	0.3659
C-Cu	6	57.7 ± 1.6	−3.0	0.3311
C-Mn	6	58.7 ± 1.2	−2.0	0.3311
C-SZCM	6	67.3 ± 6.0	+6.6	0.6864

Statistical analysis was calculated using the Gehan–Breslow–Wilcoxon test; ** indicates significance (*p* < 0.01). (a) Survival in the cisplatin group was compared with survival in the “Normal diet” group. n: sample size; SEM: standard error of the mean; normal diet: standard rodent diet; control (C): control diet prepared from scratch; C-Se: diet without selenium; C-Zn; diet without zinc; C-Cu: diet without copper; C-Mn: diet without manganese; C-SZCM: diet without selenium, zinc, copper, and manganese.

**Table 4:** The survival of mice with ovarian cancer treated with cisplatin or with the diet lacking selenium.

Treatment	n	Survival Time (Mean ± SEM; Days)	Survival Improvement vs. Control (Days)	*p* Value vs. Control
Control (a)	12	59.4 ± 1.3	-	-
C-Se (a)	12	74.1 ± 7.5	14.7	0.0207 (*)
Cisplatin (b)	4	83.5 ± 2.7	24.1	0.0061 (**)

Statistical analysis was calculated using the Gehan–Breslow–Wilcoxon test; * indicates significance (*p* < 0.05), ** indicates significance (*p* < 0.01). (a) Data from two independent experiments. (b) Data from one experiment. n: sample size; SEM: standard error of the mean; control: control diet prepared from scratch; C-Se: diet without selenium. Animals treated with cisplatin received the control diet prepared from scratch.

**Table 5:** Survival of mice with metastatic colon cancer treated with capecitabine or with diet lacking selenium.

Treatment	n	Survival Time (Mean ± SEM; Days)	Survival Improvement vs. Control (Days)	*p* Value vs. Control
Normal diet	5	30.0 ± 1.5	-	-
Capecitabine (b)	5	41.8 ± 2.7	+11.8	0.0206 (*)
Control	8	31.0 ± 1.4	-	-
C-Se	8	38.9 ± 3.8	+7.9	0.0684

Statistical analysis was calculated using the Gehan–Breslow–Wilcoxon test; * indicates significance (*p* < 0.05). (b) Survival in the capecitabine group was compared with survival in the “Normal diet” group. n: sample size; SEM: standard error of the mean; normal diet: standard rodent diet; control: control diet prepared from scratch; C-Se: diet without selenium.

**Table 6:** Survival of mice with triple-negative breast cancer treated with cisplatin or with diet lacking selenium.

Treatment	n	Survival Time (Mean ± SEM; Days)	Survival Improvement vs. Control (Days)	*p* Value vs. Control
Control	7	28.3 ± 2.2	-	-
Cisplatin	5	39.0 ± 4.7	+10.7	0.1258
C-Se	8	36.9 ± 6.9	+8.6	0.9084

Statistical analysis was calculated using the Gehan–Breslow–Wilcoxon test. n: sample size; SEM: standard error of the mean; control: control diet prepared from scratch; C-Se: diet without selenium. Animals treated with cisplatin received the control diet prepared from scratch.

## Data Availability

Data are contained within the article.
